# Managing Meningoencephalitis in Indian ICU

**DOI:** 10.5005/jp-journals-10071-23189

**Published:** 2019-06

**Authors:** Harsh Sapra, Vasudha Singhal

**Affiliations:** 1,2 Department of Neuroanesthesiology and Critical Care, Medanta – The Medicity, Gurugram, Haryana, India

## Abstract

**How to cite this article:** Sapra H, Singhal V. Managing Meningo-encephalitis in Indian ICU. Indian J Crit Care Med 2019;23(Suppl 2):S124–S128.

## INTRODUCTION

Meningoencephalitis, in simple terms, refers to the inflammation of the meninges and brain, and is considered an infectious neurological emergency. The presence of fever, altered sensorium and seizures in a patient should raise the clinical suspicion of meningoencephalitis, and the diagnosis should be confirmed expeditiously with cerebrospinal fluid (CSF) examination. Rapid diagnosis and treatment of acute meningoencephalitis with antibiotics or antiviral therapy is the key to prevent long term neurological sequelae. A thorough history, examination, laboratory analysis, and neuroimaging — together help in adequate decision making.

The syndrome of meningoencephalitis ranges from meningeal inflammation, presenting as headache, fever and nuchal rigidity, to inflammation of the brain parenchyma, leading to cortical dysfunction, and symptoms of seizures and focal deficits like aphasia and hemiparesis. The infection may be caused by bacteria such as *Streptococcus pneumonia*, *Neisseria meningitides*, *Hemophilus influenzae*, *E. coli*, *Mycobacterium tuberculosis* etc; fungi like *Cryptococcus neoformans*; parasites like *Plasmodium* and *Toxoplasma*; or viruses like *Herpes simplex* virus. The incidence of bacterial meningitis in pediatric patients has gone down drastically with the advent of Hib (Hemophilus influenza type B) and conjugate pneumocococcal vaccines, although it does not preclude infection from other strains. Tubercular meningitis (TBM), caused by *Mycobacterium tuberculosis*, is a form of subacute or chronic meningitis, that needs special consideration in the Indian scenario. The diagnosis of TBM is challenging, and so is the long term prognosis, in view of the various complications that it presents with – namely, hydrocephalus, vasculitic infarcts, elevated intracranial pressure, hyponatremia, seizures etc.

The presentation of different syndromes in meningoencephalitis is determined by the abnormalities of the host response. The infecting organism gains entry into the central nervous system (CNS) through hematogenous spread via arterial blood, or through direct extension from another site of infection such as infected bone or sinuses secondary to trauma. Foreign bodies in the CNS, such as ventriculo peritoneal shunts (VP shunts), provide the environment for bacteria to multiply.^1^ Immunocompromised states such as, diabetes, asplenia, human immunodeficiency virus (HIV) patients, patients on steroids, immunosuppressive drug therapy, radio- and chemotherapy – all predispose to CNS infections. Other risk factors include, extremes of age (neonates and elderly), crowding, contiguous infection such as otitis or sinusitis, indwelling devices such as VP shunts, recent neurosurgery or head trauma, and bacterial endocarditis in patients with prosthetic valves or intravenous drug abusers.^2^

## ETIOLOGY

The causative organism for bacterial meningitis can be predicted by the patient's age, immunocompetence, and predisposing factors.^3^ The most common bacteria causing meningitis in immunocompetent individuals are– *Streptococcus pneumoniae* and *Neisseria meningitides*. Extremes of age, both neonates and elderly, are additionally predisposed to infections by *Listeria monocytogenes* and *Streptococcus agalactiae*, along with *E. coli.* In immunocompromised patients, the commonest pathogens causing infection are *Streptococcus pneumoniae, Listeria monocytogenes,* and gram-negative bacilli, including *Pseudomonas aeruginosa.* Nosocomial meningitis is most commonly caused by resistant *Staphylococci* (methicillin resistant *Staph aureus*) and gram-negative bacilli, usually multidrug resistant, including *Pseudomonas* and *Klebsiella.* Appropriate empirical antibiotics, targeting the commonest organism for the particular patient, is the corner-stone for improving outcomes in patients with meningitis.

Fungal meningitis is usually secondary to systemic mycosis, and is most commonly caused by the *Cryptococcus* species in an immunocompromised patient. *Coccidiodes* sp. and *Histoplasma* sp. are less common causes of fungal CNS infection. *Candida* infections of the CNS are usually hospital acquired.

Encephalitis is most commonly caused by viral infections – enteroviruses (*Coxsachie* and *Echoviruses*) being the commonest (seen in ~80% of all patients with aseptic meningitis). *Herpes simplex* virus (HSV-1 infection in ~90%, remaining by HSV-2) is another frequent cause, leading to the most life threatening form of encephalitis with a high mortality and morbidity. The less common causes include mumps, HIV, cytomegalovirus (CMV), varicella zoster etc.^4^ Infections with CMV, Epstein Barr virus (EBV) and human herpes virus-6 are increasingly being seen in immunocompromised patients.

### Clinical Presentation

The classic triad of fever, stiff neck and altered sensorium are seen in <50% of all patients with meningitis. Ninety-five percent of patients will, however, have two out of the four symptoms of headache, fever, neck stiffness and altered consciousness.^5^ The occurrence of seizures indicates cortical irritation in a patient with CNS infection and is an independent predictor of mortality.^6^ Multiple cerebral infarcts secondary to vasculitis or venous thrombosis may cause cerebral edema and raised intracranial pressure (ICP). Many manifestations of meningoencephalitis may point toward the causative organism – a petechial or purpural rash in meningococcal meningitis; ataxia and labyrinthitis in *H. influenzae* meningitis; cough, weight loss and cranial nerve deficits in TBM. Systemic complications like septic shock, pneumonia and disseminated intravascular coagulation may occasionally be seen in complicated bacterial meningitis.

Clinical presentation of viral encephalitis includes a prodrome of fever, headache, myalgia, and mild respiratory infection. Altered mentation, focal neurological deficits and seizures usually follow. A herpetic rash may be seen in herpes simplex encephalitis. In immunocompromised patients with brainstem involvement, diplopia, dysarthria and ataxia may be present. Herpes simplex encephalitis, which has a predilection for the temporal and orbitofrontal lobes, may present with personality change, memory loss, confusion or olfactory hallucinations. Neonates with meningitis may present with irritability, hypotonia, decreased feeding, vomiting, listlessness and seizures (mostly focal).

### Management

#### Emergency Management

Patients presenting with symptoms and signs suggestive of meningoencephalitis should be triaged on priority in order to reduce mortality and long term disability. The ABC of resuscitation should be followed. The patient's airway is assessed for the need for intubation, and vitals, which include heart rate, blood pressure, respiratory rate, temperature, oxygen saturation, and Glasgow Coma Scale (GCS) are noted. A quick blood glucose check is followed by intravenous cannulation for immediate fluid resuscitation. Blood samples are withdrawn for hemogram, coagulation profile, renal and liver function tests, serum lactate levels, and most importantly, blood cultures. Inotropes (vasopressors) may be used to support blood pressure if the patient does not respond to a fluid challenge.

In patients suspected with bacterial meningitis, broad spectrum parenteral antibiotics with a good CSF penetration and a bactericidal action, need to be started immediately without any delay for imaging or lumbar puncture (Table 1). Delaying appropriate antibiotic treatment by even a few hours has been associated with worse outcomes in patients with bacterial meningitis.^7^ A third generation cephalosporin, like ceftriaxone (2g iv q12h) and vancomycin (1g iv q12h) are reasonable choices to begin with. Ampicillin 2g iv q4h may be added in elderly and immunosuppressed patients to cover Listeria. In nosocomial infections, antipseudomonal cephalosporins (ceftazidime or cefipime) or carbapenems (meropenem) should be considered in order to cover resistant gram negative bacteria, along with Vancomycin. A quick shot of intravenous (iv) dexamethasone 10 mg precedes the initiation of antibiotics. If herpes encephalitis is one of the differentials of the patient, antiviral therapy with iv acyclovir 10 mg/kg may be added. In immunocompromised patients with suspected fungal meningitis, liposomal amphotericin-B 3-5 mg/kg iv daily is initiated.

The stepwise management of meningitis is as follows – first step, corticosteroids; second step, empiric antibiotics; third step, imaging; fourth step, lumbar puncture; and fifth step, management of complications.

### Diagnostic Workup

CSF analysis is the key to diagnose and manage a case of meningoencephalitis. CSF analysis (via lumbar puncture (LP) / VP shunt tap) should be performed as early as feasible in all patients to guide treatment. However, two major issues that may delay a lumbar puncture include – a concern for uncal or tonsillar herniation, and the need to initiate empiric antibiotics urgently. Firstly, empiric antibiotics have to be started immediately in a patient with a clinical suspicion of infection, as CSF sterilization even with the most sensitive organisms takes atleast 4-6 hours. So, the results of CSF culture are not flawed even if the patient has been administered antibiotics in the last few hours. Secondly, a non-contrast computed tomography (NCCT) head should be done in all patients with a risk of brain herniation, such as patients with intracranial tumours, so as to rule out a raised ICP.

*Role of CNS imaging:* A CT head may help to identify patients with lesions that place them at risk of herniation following LP, and to diagnose conditions which make LP unnecessary or that would be missed if the patient's workup was limited to CSF analysis, such as a brain tumor. It has however, been debated that there is no role of performing a routine CT head in all patients suspected with meningoencephalitis, as it unnecessarily delays early antibiotic administration and CSF analysis, both of which play a very important role in improving patient outcomes.

The Infectious Diseases Society of America (IDSA) guidelines delineate criteria for the use of CT head before LP in adults with community-acquired meningitis.^8^ These include:

- Immunocompromised states, such as HIV, patients receiving immunosuppressive therapy, or post-transplant – to rule out toxoplasma encephalitis or lymphoma
- History of CNS disease (e.g., mass lesion, stroke, or focal infection)
- New onset seizure (within 1 week of presentation)
- Abnormal neurological findings such as papilledema, abnormal level of consciousness, and focal neurological deficits

What must be noted is the fact that the best predictors of when to delay LP because of risk of herniation were clinical signs of impending herniation, rather than findings on CT.

**Table 1:** Empiric antimicrobial therapy in acute bacterial meningitis

*Age group*	*Causative pathogens*	*Empirical therapy*
Infants and children	*S. pneumoniae, N. meningiditis, H. Influenzae, S. agalactiae*	Ceftriaxone 50 mg/kg iv, plus Vancomycin 15 mg/kg iv
Adults	*S. pneumoniae, N. meningiditis*	Ceftriaxone 2 g IV q 12 hrs, plus Vancomycin 15 mg/kg q 12 h
Neonates and elderly	*S. pneumoniae, N. meningiditis, L. monocytogenes*	Ampicillin 2g iv q 6h, plus Ceftriaxone 2 g IV q 12 hrs, plus Vancomycin 15 mg/kg q 12 h
Immunocompromised	*S. pneumoniae, N. meningiditis, H. influenzae*, aerobic Gram-negative bacilli	Ampicillin 2g iv q 6h, plus Ceftazidime 2 g iv q8h or Cefipime 2g iv q8h, plus Vancomycin 15 mg/kg q 12h
Nosocomial	*S. aureus, S. epidermidis,* aerobic Gram-negative Bacilli	Ceftazidime 2 g iv q8h or Cefipime 2g iv q8h or Meropenem 40 mg/kg iv q8h, plus Vancomycin 15 mg/kg q 12h

MRI brain, though not routinely performed, is the most sensitive imaging modality in patients with meningoencephalitis. It detects the presence and extent of inflammatory changes in the meninges (in the form of leptomeningeal enhancement), along with the identification of complications such as hydrocephalus, cerebral edema or stroke. Distension of the subarachnoid space with widening of the interhemispheric fissure is an early finding seen in meningitis. In HSV encephalitis, hyperintensities and diffusion restriction in the cortical and subcortical regions of bilateral fronto-temporal lobes and insula are characteristically seen. In TBM, thick basal exudates, hydrocephalus, infarcts and ring enhancing lesions or tuberculomas are typically present on MRI brain.

*CSF analysis:* A lumbar puncture is performed with a measurement of the CSF opening pressure (in the left lateral decubitus position), and CSF is sent for cytology (number and type of cells), biochemistry (glucose, protein, chloride), lactate, gram's stain, culture and sensitivity (Table 2).

Polymerase chain reaction (PCR) assay is a useful aid for the rapid detection of causative pathogens (100% sensitive and 98.5% specific). It may be used to detect bacterial DNA (*Neisseria, H. influenzae, S. pneumoniae, Streptococcus agalactiae, and Listeria*), viral meningitis panel (*Herpes simplex, Cytomegalovirus, Enterovirus, Varicella zoster*) and *Cryptococcus*. CSF adenosine deaminase (ADA), *Mycobacterium tuberculosis* PCR and nucleic acid amplification (NAA) tests help in the diagnosis of tubercular meningitis.

An elevated opening pressure (>20 cm H_2_O), with neutrophilic pleocytosis (cell count >1000/mm^3^), low CSF glucose (<2/3^rd^ of blood glucose) and raised proteins are indicative of a bacterial etiology. Positive CSF Gram's stain and cultures confirm the diagnosis of bacterial meningitis. CSF lactate concentration concentration (>4 mmol/L) has an additional diagnostic value in hospital acquired bacterial meningitis, though may lack specificity in community acquired cases. Lymphocytic pleocytosis with a low glucose and very high proteins raise the suspicion of tubercular meningitis, which can be confirmed by TB PCR and NAA tests.

Viral meningoencephalitis usually has a normal or slightly elevated opening pressure, normal to low glucose, high proteins, and a lymphocytic pleocytosis (neutrophilic predominance may be seen in very early stages). The red blood cell count in CSF may be markedly elevated in HSV-1 encephalitis, as the CSF is hemorrhagic. PCR for HSV detection is highly sensitive and specific in diagnosing this condition.

*Role of electroencephalography (EEG):* EEG is strongly recommended in any suspected case of acute encephalitis. It helps in distinguishing focal encephalitis (e.g. temporal discharges in HSV encephalitis) from generalized encephalopathy (diffuse bihemispheric slowing).

#### Definitive Management

The definitive treatment of meningoencephalitis depends upon the identification and antimicrobial sensitivity of the causative organism; taking care of the mass effect that the CNS infection may cause by means of antiedema measures; and modulation of the host immune response through steroids to minimize excessive inflammation while treating the infection.

#### Steroids

Current guidelines recommend the use of dexamethasone 10 mg iv q6h (0.15 mg/kg in children) concurrently with antibiotics in all patients suspected with acute bacterial meningitis.^9^ They help to abort the effect of inflammatory cytokines released due to bacterial lysis by the antibiotics. These inflammatory cytokines are responsible for most complications of meningitis, such as seizures, sensorineural hearing loss, venous infarcts and raised ICP. Treatment with steroids seems to lower the incidence of these complications, esp in *Streptococcus pneumoniae* infection, providing a survival benefit. They should however, be avoided in patients previously on antibiotics.

Steroids are also recommended as a part of standard therapy in tubercular meningitis to reduce inflammation and cerebral edema.^10^ They are known to reduce mortality, atleast in the short term.^11^ Dexamethasone in a dose of 0.4 mg/kg iv in adults should be continued for 4 weeks, and tapered gradually over 2–4 weeks.

In viral encephalitis, adjuvant steroids are recommended to reduce cerebral edema in the delayed phase (day 4 onward), thereby improving outcomes substantially.^12^ In early phases however (day 1–3), the use of steroid may enhance viral replication proving harmful, and should be avoided.

#### Antimicrobial Therapy

Once the results of culture sensitivity of the etiological organism are available, the ongoing antibiotics are modified. Of special concern are the multidrug resistant gram negative bacilli like the *Pseudomonas*, *Acinetobacter* and *Klebsiella*, seen mostly in nosocomial settings with infected shunt devices. These micro-organisms are often carbepenem resistant, and necessitate initiation of colistin, an antibiotic that has a poor CSF penetration. As a result, the infection responds poorly to systemic therapy alone. An alternative to this is intrathecal/ intraventricular administration of the antibiotic in order to achieve bacterial eradication. The combination of intravenous plus intraventricular colistin therapy may improve outcomes in patients with meningitis due to multi-drug resistant infections.^13^ The dose of intraventricular colistin ranges from 5-20 mg preservative free solution daily. Other antimicrobials administered via intraventricular route include – vancomycin, amikacin, polymyxin B etc. In patients with infected shunts, the best practice is to remove, replace or externalize the infected hardware as appropriate.

**Table 2:** CSF picture in meningitis

*CSF parameter*	*Normal*	*Bacterial meningitis*	*Viral meningitis*	*Tubercular meningitis*	*Fungal meningitis*
Opening pressure	5–20 cm H_2_O	Elevated	Normal	Normal	Normal or elevated
Cell count	<5 cells/mm^3^	>1000/mm^3^	<1000/mm^3^	100–500/mm^3^	100–500/mm^3^
Cell predominance	Nil	Neutrophils	Lymphocytes	Lymphocytes	Lymphocytes
CSF glucose	>2/3rd of serum glucose	Low	Normal	Low	Low
CSF proteins	<45 mg/dL	Elevated	Normal/ elevated	Elevated	Elevated

In TBM, the Infectious Diseases Society of America (IDSA) and the American Thoracic Society recommends an antitubercular regimen similar to that followed for pulmonary TB – an intensive 2-month phase with four drugs (isoniazid/rifampicin/pyrazinamide/ ethambutol) followed by a continuation phase of upto 10 months with isoniazid and rifampcin.^14^ Of concern in the treatment of TBM is a rise in the multidrug resistant (MDR) TB, resistant to both isoniazid and Rifampicin. The WHO guidelines state the use of atleast five effective drugs initially in the treatment of MDR TBM. These include a fluoroquinolone and an injectable second line agent like amikacin, capreomycin, kanamycin, ethionamide, cycloserine or linezolid, with treatment lasting for 18–24 months.^15^

Most cases of viral encephalitis are self-limiting and the treatment is largely supportive. This includes symptomatic treatment of fever, headache, nausea; airway protection if needed; appropriate fluid and electrolyte optimization; and management of complications such as raised ICP and seizures.^16^ Specific antiviral therapy for viral encephalitis is generally limited to disease caused by the herpes simplex virus. Early initiation of acyclovir 10 mg/kg iv q8h in patients suspected with HSV encephalitis (within 2 days of onset) reduces the likelihood of death and serious sequelae. The treatment should be continued for 14–21 days. During treatment, adequate hydration needs to be maintained, as acyclovir is known to cause a crystal induced nephropathy. CMV encephalitis, mostly seen in transplant recipients, is treated with a combination of ganciclovir (5 mg/kg iv q12h) and foscarnet (60 mg/kg iv q8h).^17^ Both ganciclovir and foscarnet require careful monitoring for renal dysfunction, bone marrow suppression and seizures.

The treatment regimen for fungal meningitis comprises of an initial induction phase with amphotericin (liposomal amphotericin 3-5 mg/kg/day) for 2-6 weeks depending upon the clinical response, followed by a consolidation phase with fluconazole (400 mg/day) for upto 8 weeks or even longer. Flucytosine (100 mg/kg/day orally in four divided doses) is often added to amphotericin-B during the induction phase for the treatment of *Cryptococcal*, *Candida* and *Aspergillus* infections. The use of intrathecal amphotericin-B in conjunction with systemic amphotericin and flucytosine, in invasive fungal CNS infections that have not responded to systemic therapy or those who have relapsed, has also been reported. The dose for intrathecal amphotericin-B ranges from 0.01-1.5 mg and intervals range from daily to weekly.^18^ Voriconazole (6 mg/kg iv q12h for two doses, followed by 4 mg/kg q12h) may be a promising therapy in the management of susceptible CNS fungal infections, esp invasive aspergillosis.

### Management of Complications

Acute hydrocephalus, most commonly seen in tubercular or cryptococcal meningitis, needs repeated lumbar punctures or a ventricular drain for acute management. Hyponatremia, caused by either salt wasting or syndrome of inappropriate anti-diuretic hormone (SIADH), may lead to an altered sensorium and should be carefully managed. Seizures may be seen in 15% cases of acute meningitis, and many a times, an EEG may be indicated to detect non-convulsive status epilepticus causing a fluctuating level of consciousness in these patients. Sensorineural hearing loss is a common complication seen with bacterial meningitis. Acute stroke due to vasculitis, venous thrombosis and cerebral edema causing a raised ICP are frequently seen in complicated meningoencephalitis. Appropriate anti-edema measures, including osmotic diuretics, are indicated to prevent neurological sequelae.

### Tropical Disease-associated Encephalitis

In India, acute encephalitis syndromes (AES) related to the seasonal outbreak of tropical diseases occur regularly, causing substantial mortality especially in the pediatric age group. In over 50% of the patients with AES, the source or causal agent goes unrecognized. The treatment is largely supportive.

The commonest etiological agent with a high endemic burden in India is the mosquito-borne flavivirus – *Japanese encephalitis*. The detection of IgM antibodies to the Japanese Encephalitis virus in the CSF or serum may confirm the diagnosis. MRI brain typically shows thalamic hyperintensities on T2 weighted imaging. The basal ganglia, brainstem and cerebellum may also show focal abnormalities. This disease has a high case fatality of ~25–30%, and a permanent neurological sequalae rate of ~50%. The incidence of Japanese encephalitis has however decreased in the recent years due to successful vaccination programmes in the 181 endemic districts of India, including eastern UP, Bihar, and West Bengal.^19^ More recently, many other viruses like the enterovirus, Chandipura virus, Nipah virus, Zika virus, dengue virus, Chikungunya virus etc., have emerged and have been implicated in the outbreaks of AES in the Indian subcontinent.^20^

*Dengue*, classically thought to be a non-neurotropic virus, is now known to cause isolated encephalitis. The detection of dengue viral RNA and IgM antibodies in the CSF may be disease-course dependent, and may not always be seen. If a patient presents with encephalitic signs and symptoms (focal neurological deficits, convulsions etc.) in endemic areas, and show a lymphocytic pleocytosis in the CSF with serum positive for dengue IgM antibodies, a differential diagnosis of dengue encephalitis should be considered. Involvement of bilateral thalami, pons and medulla may be seen on imaging in these patients. Petechial hemorrhages and diffuse cerebral edema may also be seen in some.

*Chikungunya virus*, a reemerging alphavirus infection, transmitted to humans by Aedes mosquito, is associated with encephalitis at extremes of age, with a high proportion of patients discharged with permanent disabilities. Detection of viral RNA in the CSF by PCR, or CSF IgM antibody by ELISA is considered diagnostic. The imaging studies may show cingulate gyrus and limbic system involvement.

*Zika virus*, another mosquito-borne flavivirus causing encephalitis, is endemic to central India. The symptoms of Zika virus are similar to dengue and chikungunya. Reverse transcription (RT) PCR of acute phase serum samples is the test of choice. There is a possible association of microcephaly in newborn babies by mother-to-child transmission, and peripheral nervous syndromes especially Guillain Barre Syndrome (GBS) in adults.

In recent years, the *Chandipura virus* (a rhabdovirus, vector – sandfly) has emerged as an encephalitic pathogen, causing a number of outbreaks in different parts of the country, including Gujarat, Maharashtra and Andhra Pradesh. Chandipura virus, like the *rabies virus* (another rhabdovirus affecting the CNS), enters the brain by retrograde movement from the peripheral or olfactory neurons, causing severe neurodegeneration. Early treatment with mannitol to reduce cerebral edema may be life saving.

*Nipah virus infection*, transmitted through pigs and fruit bats, is a public health concern especially in southern states of India, owing to its high mortality. Real time PCR from body fluids and antibody detection remain the main diagnostic tests. Multiple small white matter lesions may be seen on MRI. There are no vaccines or drugs for Nipah virus encephalitis, and intensive supportive care is all that can be offered.

None of the viral encephalitic syndromes discussed here have a specific treatment. Intensive care support with maintenance of perfusion, hydration and oxygenation is the common goal of therapy. Control of intracranial hypertension and management of seizures should be done to improve survival. The long-term morbidity remains very high.

## CONCLUSION

Acute meningoencephalitis is a devastating disease, which needs early recognition and prompt treatment in order to improve mortality and morbidity. Newer diagnostic and treatment modalities present a promising picture, although the disease burden still remains high.
